# Oligomerization-Dependent Beta-Structure Formation in SARS-CoV-2 Envelope Protein

**DOI:** 10.3390/ijms232113285

**Published:** 2022-10-31

**Authors:** Wahyu Surya, Jaume Torres

**Affiliations:** School of Biological Sciences, Nanyang Technological University, 60 Nanyang Drive, Singapore 637551, Singapore

**Keywords:** envelope protein, SARS-CoV-2, ion channel, Fourier-transform infrared spectroscopy, analytical ultracentrifugation, conformational change, scission mechanism

## Abstract

The severe acute respiratory syndrome coronavirus 2 (SARS-CoV-2) is responsible for the current COVID-19 pandemic. In SARS-CoV-2, the channel-forming envelope (E) protein is almost identical to the E protein in SARS-CoV, and both share an identical α-helical channel-forming domain. Structures for the latter are available in both detergent and lipid membranes. However, models of the extramembrane domains have only been obtained from solution NMR in detergents, and show no β-strands, in contrast to secondary-structure predictions. Herein, we have studied the conformation of purified SARS-CoV-2 E protein in lipid bilayers that mimic the composition of ER–Golgi intermediate compartment (ERGIC) membranes. The full-length E protein at high protein-to-lipid ratios produced a clear shoulder at 1635 cm^−1^, consistent with the β-structure, but this was absent when the E protein was diluted, which instead showed a band at around 1688 cm^−1^, usually assigned to β-turns. The results were similar with a mixture of POPC:POPG (2-oleoyl-1-palmitoyl-sn-glycero-3-phosphocholine/3-glycerol) and also when using an E-truncated form (residues 8–65). However, the latter only showed β-structure formation at the highest concentration tested, while having a weaker oligomerization tendency in detergents than in full-length E protein. Therefore, we conclude that E monomer–monomer interaction triggers formation of the β-structure from an undefined structure (possibly β-turns) in at least about 15 residues located at the C-terminal extramembrane domain. Due to its proximity to the channel, this β-structure domain could modulate channel activity or modify membrane structure at the time of virion formation inside the cell.

## 1. Introduction

The causative agent of the current COVID-19 pandemic is severe acute respiratory syndrome coronavirus 2 (SARS-CoV-2, or SARS-2 for short), a β-coronavirus in the subgenus sarbecovirus with 79% identity to SARS virus [1] that not only causes severe respiratory disease but also affects the CNS (anosmia), GI tract (diarrhea), clotting system (thrombosis), heart (arrhythmias), immune system (cytokine storm), and liver and kidney (damage) [2,3,4,5,6,7]. The mortality caused by SARS-2 is lower than that of the severe acute respiratory syndrome (SARS) or Middle East respiratory syndrome (MERS) coronaviruses [8,9,10], but it is far more contagious [11,12]. Since the SARS-2 pandemic began, almost 600 million have been infected, and more than 6.4 million have died. Thus, the world is in urgent need of antivirals and needs to prepare rapid-response strategies for future coronavirus pandemics.

In SARS, the envelope E protein is a well-known virulence factor [13]. SARS E protein is 76 amino acids long and localizes to the endoplasmic reticulum–Golgi intermediate compartment (ERGIC) of infected cells [14], where the C-terminus is cytoplasm-oriented [14]. Our lab and others have shown that E protein forms ion channels [13,14,15,16,17,18,19,20,21,22,23,24,25,26], using full-length E (E-FL), its transmembrane domain (E-TM), or a truncated form (E-TR, residues 8–65) [18,19,26]. The link between pathogenicity and channel activity was triggered after the discovery of channel-inactivating mutations at the transmembrane domain (TMD) of the E protein, N15A, and V25F [15]. When these were introduced into a mouse-adapted SARS virus, they led to mice survival, which exhibited reduced lung edema and lower proinflammatory cytokine levels [13]. In contrast, the wild-type E protein led to the inflammasome activation and elevated production of IL-1β, lung epithelial cell damage, and death [13]. Escape mutations were observed only in the TMD of the E protein, which is responsible for channel activity. Viruses bearing these mutations regained virulence, and, concomitantly, the E protein regained ion channel activity [13].

**Figure 1:**
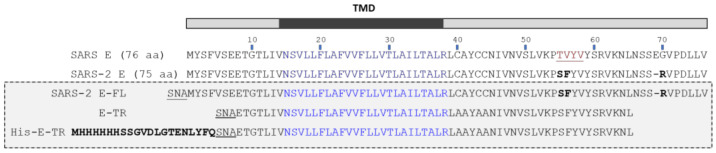
Comparison of SARS E sequences. Sequence comparison between SARS E (accession number P59637) and SARS-2 E (accession number QHZ00401). Transmembrane domain (TMD) is shown in blue, whereas residues previously mutated to Ala (TVYV) in SARS E [18] are shown in red. SARS-2 E only differs from SARS E by three residues (bold) and one deletion. The samples used in the present paper (gray rectangle) correspond to SARS-2: full length (E-FL), truncated (E-TR), or truncated with a His-tag (His-E-TR). Both E-FL and E-TR contain additional three N-terminal residues (SNA), arising from the N-terminal fusion tag that is cleaved.

It has been shown that Ca^2+^ fluxes through the E-channel are involved in SARS pathogenicity via activation of the NOD-, LRR-, and pyrin-domain-containing protein 3 (NLRP3) inflammasome [20], similar to other systems [27,28,29]. Although the biology of the E protein of SARS-2 has not yet been examined in detail, the sequences of the E-protein in SARS (76 residues) and SARS-2 (75 residues) are almost identical, with the exception of one deletion and two conservative substitutions (Figure 1). Thus, based on this similarity, it is likely that SARS-2 E can serve as a target for therapeutic approaches to tackle COVID-19.

The structure of E-TM (identical in SARS and SARS-2) is completely α-helical, as shown by studies in detergent dodecylphosphorylcholine (DPC) [25], lipid membranes formed by 1,2-dimyristoyl-sn-glycero-3-phosphocholine (DMPC) [23], and, more recently, using solid-state NMR and endoplasmic reticulum–Golgi intermediate compartment (ERGIC)-like membranes [30]. Information regarding the extramembrane domains of SARS E has been obtained by solution NMR using a DPC:SDS mixture [18] or LMPG (1-myristoyl-2-hydroxy-sn-glycero-3-phospho-(1′-rac-glycerol)) detergent [31]. In the latter, E-TR (65 residues) showed 27 residues, strictly α-helical (https://swissmodel.expasy.org/ (accessed on 15 October 2022)), and 9 more were assigned to the 3–10 helices; therefore, a total of 36 residues are ‘helical’ (63%). Other residues (37%) are variously assigned to the coil, bend, or loops and turns. However, no regions of the extended β-strand were detected, despite the predicted presence of a β-turn-β motif in the C-terminal region around a conserved Pro residue. This β-structure region is predicted for the E protein in SARS, SARS-2, and other coronaviruses [18].

Although these solution NMR studies in detergent showed no evidence of a β-structure for E-FL or E-TR, a distinctive shoulder corresponding to the β-structure around 1635 cm^−1^ (assigned to β-structure) was observed in the Fourier-transform infrared (FTIR) amide I band of SARS E in DMPC membranes [18]. This was accompanied by another shoulder centered around 1685 cm^−1^, typically assigned to the β-turns [32,33]. The mutation of residues 56–59 (formed by β–branched Val or bulky Tyr) to small-side-chain Ala (see Figure 1, TVYV) eliminated these two features, suggesting the presence of β-structure and β-turns conformation in a small part of the E population or a small part of the E protein. The changes observed were consistent with the expected increase in helicity at the mutated part of the molecule [34], and we proposed that the existing β-structure may have been in dynamic equilibrium with a more abundant α-helical form. We also showed that a synthetic peptide encompassing this putative β-hairpin region (residues 46–60) folds as a β-structure and absorbs at 1635 cm^−1^, is completely resistant to hydrogen/deuterium (H/D) exchange, and has a high tendency to aggregate in solution, forming amyloid fibers [18,35]. However, it was not clear what caused this β-structure to appear in the isolated peptide but not in the context of E-FL or E-TR in detergents.

Herein, we have tested the hypothesis that, rather than being dependent on the solubilization medium (i.e., detergent vs. lipid membranes) or type of lipid (e.g., polarity, chain length), formation of β-structure in E protein is simply dependent on channel formation, i.e., oligomerization. We conclude that the structural models obtained in detergents cannot be explained solely on account of the high micelle curvature or different detergent physico-chemical environments. Given that the structure of the E protein must be solved at a high resolution in its full-length form and in a variety of environments for virtual drug screening, these are critical results that should guide future efforts in this direction.

## 2. Results

### 2.1. Formation of β-Structure in SARS-2 E-FL in ERGIC and PC:PG Membranes

**Figure 2:**
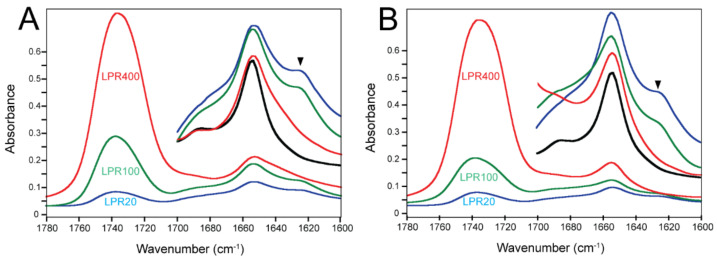
Infrared spectra corresponding to E protein in lipid membranes. Spectra of E-FL in ERGIC lipids (**A**) and in the mixture POPC:POPG (**B**) at LPR20 (blue), LPR100 (green), and LPR400 (red). The same color coding is used in the figures below. The insets show the enlarged amide I region after subtraction of the lipid component (see Figure 3A). The spectrum of the 100% α-helical E-TM (black line) is also shown for comparison. The black arrow indicates the absorption band of β-structure.

To test if lipid composition or concentration is a strong determinant of secondary structure in the full-length E protein (E-FL, Figure 1), we reconstituted the latter in negatively charged lipid membranes that mimic the environment of E protein in the cell, at three lipid-to-protein (LPR) molar ratios: 20, 100, and 400 (Figure 2). The lowest LPR of 20 (highest protein concentration) is the ratio used in a recent solid-state NMR (ssNMR) paper that studied the transmembrane domain of the E protein (E-TM) [30]. At this low LPR, the E protein is expected to be pentameric, as shown previously using analytical ultracentrifugation sedimentation equilibrium data (AUC-SE) [18,19,26] and ssNMR [30], whereas dilution of the E protein to LPR400 is expected to form only monomers.

In ERGIC lipids (Figure 2A), the amide I band of E-FL was centered at ~1654 cm^−1^, which is assigned to the α-helix [32]. However, conditions LPR20 and LPR100 also produced an evident shoulder at ~1622 cm^−1^, consistent with the β-structure (Figure 2A, inset). This shoulder disappeared after dilution at LPR400. For comparison, the amide I spectrum of E-TM, which is completely α-helical in any of these conditions, is also shown (black trace).

In a 4:1 mixture of POPC and POPG (PC:PG mixture) (Figure 2B), the results were essentially identical to those obtained in ERGIC lipid; the shoulder corresponding to the β-structure was evident at LPR20 and LPR100 but not at LPR400. This suggests that the precise lipid composition may be less important than the concentration of the E protein in the lipid phase.

**Figure 3:**
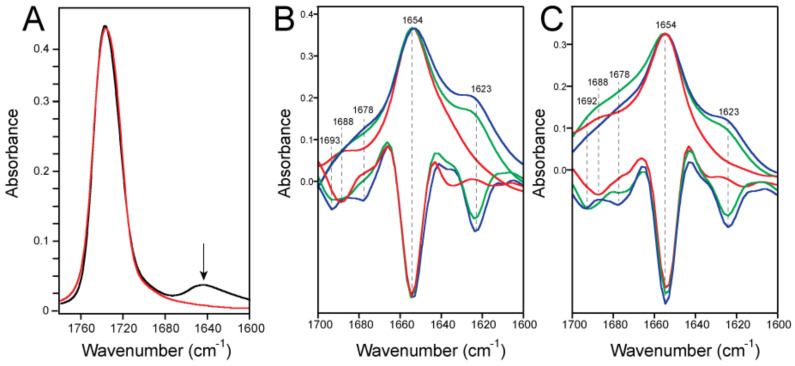
The amide I band corresponding to E-FL at various LPRs. (**A**) Spectra of lipid-only ERGIC mixture (black) or POPC:POPG (4:1) (red). The arrow indicates a small contribution of ERGIC lipids in the amide I region; this contribution was subtracted from the protein amide I; **(B-C)** amide I band of E-FL in ERGIC lipids (**B**) or in the mixture POPC:POPG (4:1) (**C**). Spectra are color-coded according to Figure 2. In each panel, the spectra are overlapped with the corresponding second-derivative spectra.

The insets of Figure 2 show amide I after removing any lipid contribution, whereas the spectra of ERGIC and PC:PG mixtures without any protein are shown in Figure 3A. The mixture of PC:PG does not absorb in amide I, whereas ERGIC lipids have a very small and broad contribution that vanishes when the lipid spectrum is subtracted from the spectrum containing the protein.

The amide I shape was examined in more detail using the second derivative to enhance the component bands (Figure 3B–C). In ERGIC lipids at LPR20, the band corresponding to β-structure was centered at 1623 cm^−1^, the band corresponding to α-helix was centered at 1654 cm^−1^, and smaller bands could be identified at 1678 and 1693 cm^−1^ (Figure 3B). At LPR400, the 1623 cm^−1^ band was not present, but a small band was observed at 1688 cm^−1^ instead. This same pattern could be observed in the PC:PG membranes (Figure 3C). Thus, at LPR400, the band centered at 1623 cm^−1^ was absent in both lipid compositions.

### 2.2. Formation of β-Structure in E-TR in ERGIC and PC:PG Membranes

**Figure 4:**
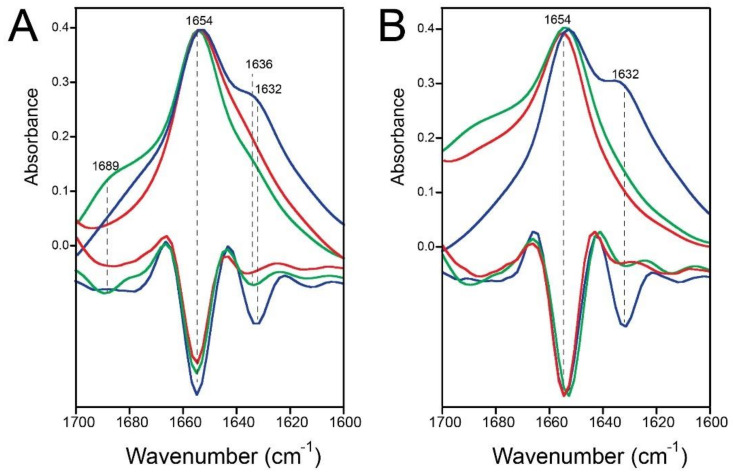
The amide I band corresponding to E-TR at various LPRs. (**A**,**B**) Amide I band of E-TR (8–65) in ERGIC lipids (**A**) or in the mixture POPC:POPG (4:1) (**B**). Spectra are color-coded according to Figure 2. In each panel, the spectra are overlapped with the corresponding second-derivative spectra.

To identify the region corresponding to this β-structure formation, we tested a truncated form of the E protein, E-TR (61 residues long, see Figure 1). This construct lacks 7 (1–7) residues at the N-terminal domain and 10 residues at the end of the C-terminal domain (66–75). At first glance, the amide I of E-TR in ERGIC lipids is similar to that of E-FL, with an amide I band centered at 1654 cm^−1^ and a clear shoulder corresponding to the β-structure at LPR20 (Figure 4A). However, in contrast to E-FL, this shoulder was only observed at LPR20 but not at LPR100 (or LPR400). In PC:PG, the results were the same as in the ERGIC lipids (Figure 4B). In addition, this shoulder was significantly blue-shifted by about 10 cm^−1^ (1632 cm^−1^), consistent with a β-structure motif, hydrogen-bond pattern, or environment different from the one formed by E-FL. Since the TM domain is known to be α-helical in lipid membranes [23,30], and the remaining extramembrane portion of the N-terminus in E-TR is too short to fully account for these changes, the domain forming the β-structure must be located within the extramembrane C-terminal domain that encompasses, approximately, residues 38 to 65. While a peptide corresponding to the last ~15 C-terminal residues in SARS-CoV E, predicted to form a random coil, adopted random coil structure in solution [18], peptides 46–60, predicted to fold as β-strands, folded experimentally as β-structure [18,35].

### 2.3. E-FL Forms Tighter Oligomers Than E-TR in Detergent

We hypothesized that, since the shoulder is at ~1620–1635 cm^−1^ and assigned to the β-structure, only appearing at a higher E protein concentration (low LPR), its presence may correlate with oligomer formation. Further, if this hypothesis is correct, since E-FL has a shoulder at both LPR20 and LPR100, whereas E-TR only has one at LPR20, the monomer–monomer affinity in E-FL must be higher than in E-TR.

We tested this hypothesis using analytical ultracentrifugation (AUC) in detergent C14-betaine. However, a direct comparison of E-FL with E-TR was not possible because the latter was very insoluble in this and the other detergents compatible with AUC. The maximum concentration that could be attained was 20 µM, which, when mixed with detergent, only produced monomers (not shown). Given this limitation, we compared E-FL (78 aa) with His-tagged E-TR (His-E-TR, 82 residues) (see Figure 1), which showed far better solubility.

**Figure 5:**
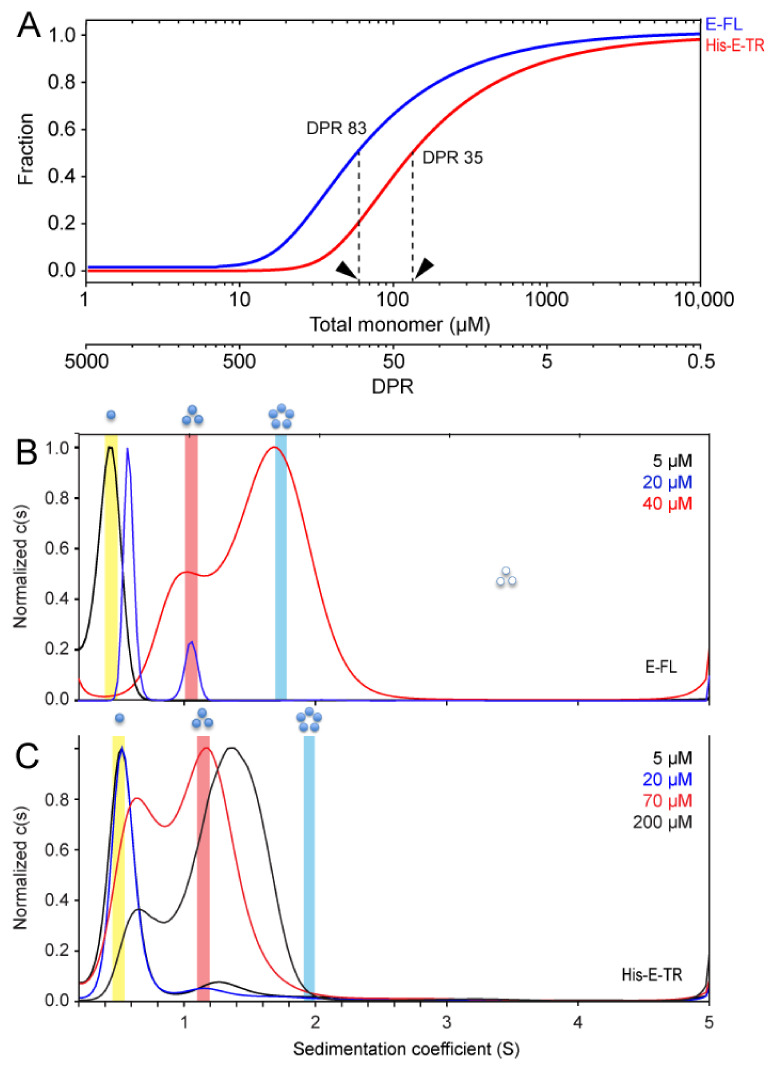
Sedimentation results of E-FL and E-TR in C14-betaine. (**A**) Fraction of the sample in the form of pentamers (y-axis), in the populations of E-FL (blue) and His-E-TR (red), as a function of either total monomer or DPR, in logarithmic scale. These data were extracted from the Ka obtained from the AUC-ES data of E-FL (Log10 Ka = 17.4) and His-E-TR (Log10 Ka = 16). The arrows indicate the DPR required for 50% pentameric formation in each sample; (**B**,**C**) c (s) plots normalized to max c (s) of E-FL (**B**) and His-E-TR (**C**) in 5 mM C14SB at the concentrations indicated. In E-FL, the concentrations are equivalent to a DPR of 1000, 500, and 125. In E-TR, they are equivalent to a DPR of 1000, 250, 71, and 25.

The AUC sedimentation equilibrium (AUC-SE) of these two constructs has been performed previously in C14-betaine [18,19]. Using the reported affinity constant (K_a_) values, the mid-point of pentamer formation is at a detergent-to-protein ratio (DPR) of 85 in E-FL and 35 in His-E-TR (Figure 5A). Therefore, 50% pentamerization is obtained at a more diluted protein concentration in E-FL than in His-E-TR; i.e., E-FL forms more stable oligomers than His-E-TR, consistent with our hypothesis.

We then used AUC in the sedimentation velocity mode (AUC-SV). In this case, stronger oligomerization is indicated by the direct observation of faster-sedimenting species. The sedimentation coefficient, S, of the various oligomeric forms can then be predicted (see Section 4). In the plots of Figure 5B–C, the calculated S-range corresponding to hypothetical monomers, trimers, and pentamers are indicated with yellow, red, and blue bands, respectively.

For E-FL (Figure 5B), at 5 μM only one band in the c (s) plot was present, consistent with the monomers. At 20 μM, the main band shifted to higher S, and a small band appeared at ~1.1 S, consistent with the homotrimers. At 40 µM, a large (~80%) proportion of E-FL shifted to S = 1.8, consistent with the pentamers. In contrast, in His-E-TR (Figure 5C) at an even higher concentration (70 μM), about 60% of the sample was centered at 1.15 S, predicted to be the trimers. Even at 200 µM, no protein was present at S > 1.9 (pentamers). Overall, these results show that E-FL has a higher tendency to form pentamers than His-E-TR in detergent C14-betaine. Therefore, if our main hypothesis is correct, His-E-TR should behave similar to E-TR in the experiments shown in Figure 2, Figure 3 and Figure 4, i.e., with the β-structure (band at ~1620–1635 cm^−1^) at LPR20 but not at LPR 100 or LPR400.

**Figure 6:**
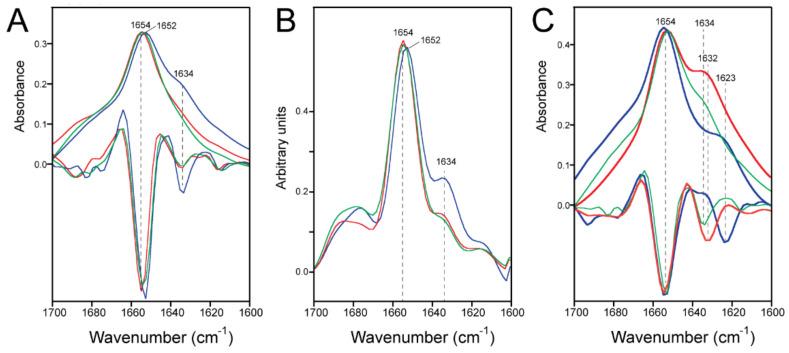
The amide I band corresponding to His-E-TR at various LPRs. (**A**) Amide I band of His-E-TR in ERGIC lipids; (**B**) mild Fourier deconvolution (bandwidth 23 cm^−1^, narrowing factor 1.5), of the spectra shown in (**A**); (**C**) comparison of amide I spectra at LPR20 for E-FL, E-TR, His-E-TR, and their corresponding second derivatives.

Thus, we repeated the experiments in Figure 2A using His-E-TR in ERGIC lipids. Indeed, similar to E-TR, an obvious shoulder at 1634 cm^−1^ was observed for LPR20 but not for LPR100 or LPR400 (Figure 6A), which is also clear in the deconvolved spectra (Figure 6B). A comparison between the LPR20 condition for E-FL, E-TR, and His-E-TR shows the redshift of this shoulder in the E-FL sample compared to E-TR and His-E-TR (Figure 6C).

### 2.4. Quantification of the β-Structure Contribution

**Figure 7:**
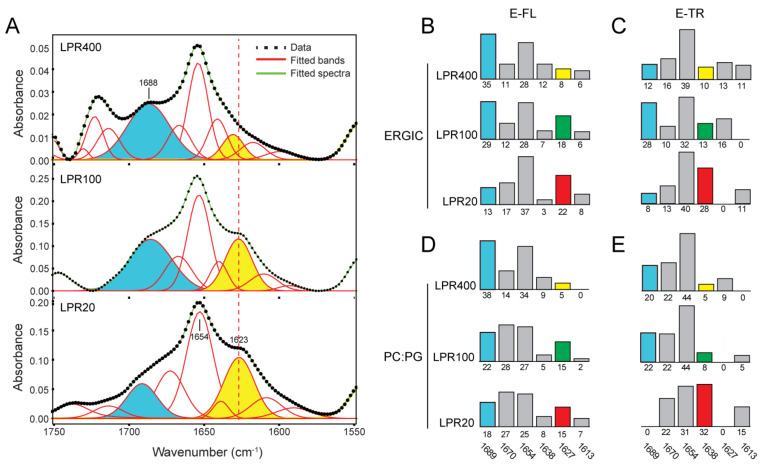
Amide I analysis of E-FL. (**A**) Representative curve-fitting of the amide I band of E-FL in ERGIC lipids at three LPRs indicated. The band assigned to β-structure is highlighted in yellow and the band at 1688 cm^−1^ in blue; (**B**–**E**) histograms representing areas under each amide I band used in the curve-fitting. The numbers at the bottom represent the center of each band (in wavenumbers), and the numbers next to the axis are the area percentages. Percentages are shown for E-FL in ERGIC (**B**) and mixture PC:PG (**C**). The band representing β-structure is shown in red (LPR20), green (LPR100), and yellow (LPR400), whereas the band at higher frequency is shown in blue, as in panel (**A**); (**C**,**D**) are same as (**B**,**C**), for E-TR.

We tried to quantify the contribution of this β-structure at ~1620–1635 cm^−1^ by curve-fitted the original amide I spectrum (Figure 7, see Section 4). For E-FL (78 aa), the area percentage of this band was ~22% at LPR20 (Figure 7B), which is maintained at LPR100 (18%) and becomes much lower at LPR400 (8%). For E-TR (61 residues) (Figure 7C), this percentage was ~28% at LPR20 and fell to 13 and 10% for LPR100 and LPR400, respectively. The higher percentage at LPR20 observed in E-TR is consistent with it being shorter than E-FL. Although this method of quantification is approximate, these results suggest that this β-region should encompass approximately 15 residues and certainly be located in the C-terminal tail.

We also note that this increase in the β-structure does not seem to occur at the expense of the α-helical content (1654 cm^−1^), since the latter did not change significantly at the different LPRs (Figure 7B–C). Moreover, the minima corresponding to the amide II (1550 cm^−1^, representing total protein content) and the α-helix (1654 cm^−1^) in the second derivative spectra of the samples of LPR20 and LPR400 showed a constant ratio (not shown). Thus, we believe that the increase at ~1620–1635 cm^−1^ seems to be at the expense of a band at higher wavenumbers, at around 1680–1690 cm^−1^. The origin of this absorption is uncertain, although it is usually assigned to the β-turns [32,33].

## 3. Discussion

In this paper, we have tested the hypothesis that the formation of the β-structure in SARS-2 CoV E is triggered by oligomerization. Current models of the extramembrane domains of E protein do not show any β-structure, although they were obtained using solution NMR in detergents. Additionally, in this paper, the protein–detergent ratio was rather low, to prevent sample aggregation. In a previous report, we showed some β-structure present in lipids at LPR50 [18] and that a ‘helix-inducing’ four Ala substitution at the C-terminal domain not only reduced the intensity of the amide I band in the 1620–1635 cm^−1^ region but also around 1685 cm^−1^, suggesting the coexistence of the β-turns and β-strands around the affected C-terminal region near Pro54. We propose that the formation of oligomers, which certainly takes place at LPR20, converts this region to the β-strands that are observable at 1620–1635 cm^−1^.

**Figure 8:**
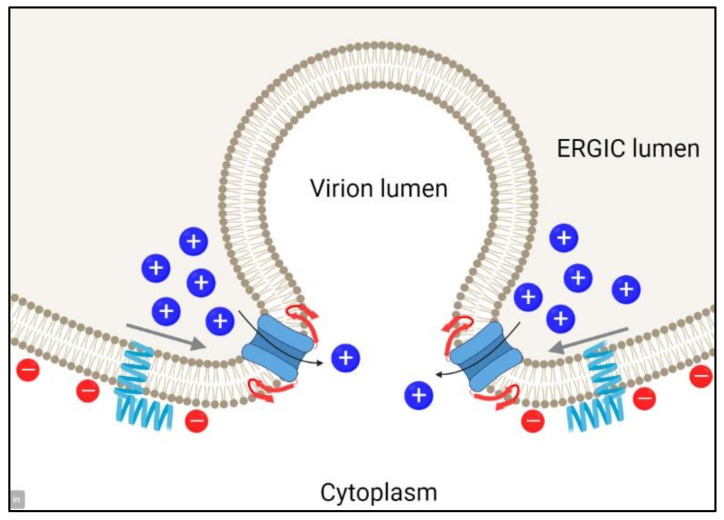
Schematic representation of the possible role of SARS-2 E protein in neck scission. The drawing represents the formation of a virion inside the lumen of the ERGIC. E protein is represented schematically by α-helical fragments (blue) when monomeric, whereas migration (arrows) and clustering into convex regions of the membrane trigger a conformational change at the C-terminal domain (red), which may insert in cis or trans (opposite) into the membrane, contributing to membrane destabilization. Ca^2+^ ions are represented by blue dots and lipid negative charges by red dots. Created with BioRender.com.

With this interpretation, one can propose a model where increased protein density may come about in the cell, triggered by the high curvature. In a recent paper, Kuzmin et al. [36] performed coarse-grained molecular dynamics simulations of the E protein monomer in model ERGIC lipids. Interestingly, the cytoplasmic domain of E protein induced curvature in the model membranes. After 1 µs, both the monomeric and oligomeric forms showed a tendency for the C-terminal tail to cluster in the regions of high convexity. The regions of high convexity are present at the time of virion formation, before scission from the ERGIC compartment (Figure 8), and these could constitute the regions of high concentration of the E protein.

Influenza A M2, another viroporin from an enveloped virus, mediates ESCRT-independent membrane scission [37]. Therefore, it is tempting to speculate that SARS-2 E protein has a similar role. Our data suggest that clustering of the E protein at these highly convex regions would facilitate the formation of pentameric channels, with concomitant formation of β-harpins at the C terminal domain. This predicted β-structure domain is predicted to be rather hydrophobic in SARS and also in other coronaviruses and may insert in the membrane, possibly interacting with the channel domain, or in adjacent membranes, helping to accomplish the scission step. The charge repulsion between opposing negatively charged ERGIC leaflets could be cancelled by the efflux of Ca^2+^ ions from the ERGIC to the cytoplasm [20], following a three orders-of-magnitude gradient.

Overall, these results suggest that future experiments should be performed to delineate the sequence requirements in the extramembrane domains of the E protein to accomplish the potential effects on the membranes, inducing high curvature or scission or modulating channel activity. These results are also important in the future search for E protein channel inhibitors, since the possible contribution of the extra membrane domains to the channel structure must be taken into account.

## 4. Materials and Methods

**SARS-CoV E protein constructs.** A full-length version, a truncated version, and a transmembrane peptide of SARS-CoV-2 E were used in this work. Full-length SARS-CoV-2 E protein (E-FL) and truncated E (His-E-TR) were expressed in *E. coli* and purified as described previously [18]. Where desired, the His-tag was removed from His-E-TR by TEV protease cleavage, prior to RP-HPLC purification, resulting in E-TR. Transmembrane peptide (residues 8–38, E-TM) was synthesized as described previously (Torres et al. 2007). As in previous work, in both E-FL and E-TR, all three native cysteines (C40, C43, C44) were mutated into alanines. The identity and purity of peptide fractions were confirmed by SDS-PAGE and MALDI-TOF MS.

**Fourier-transform infrared (FTIR) spectroscopy.** FTIR spectra was collected on peptides reconstituted in ERGIC lipid mixture (POPC:POPE:bovine PI:POPS:cholesterol = 45:20:13:7:15 molar ratio) or in negatively charged lipid mixture (POPC:POPG = 4:1 molar ratio). All lipids were purchased from Avanti Polar Lipids (Alabaster, US). E protein samples were reconstituted in lipid bilayer by dissolving a mixture of lipids in chloroform and lyophilized peptide in TFE at 20:1, 100:1, and 400:1 molar ratios. The peptide-lipid mixture was dried into a thin film under a N_2_ stream and further dried in vacuum overnight. Proteoliposomes were prepared using the film rehydration method [38] at 50 µM lipid concentration in water by vortexing, sonicating, and 7 cycles of freeze-thawing. A lipid-only sample was prepared separately, in the same way. The samples were deposited and dried on a 10-reflections ZnSe attenuated total reflection (ATR) accessory (Piketech, Madison, Wisconsin, USA). FTIR–ATR spectra were collected on a Nicolet Nexus 560 spectrometer (Nicolet, Madison, WI, USA), purged with CO_2_/H_2_O-free air, and equipped with an MCT/A detector cooled with liquid nitrogen. At least 200 interferograms were collected and averaged per spectra.

The amide I band envelope was decomposed into its components using a combination of peak fitting and second-derivative calculation [39]. Briefly, the spectra were smoothed with a 9 cm^−1^ window and the second and fourth derivatives of the spectra were calculated using OMNIC. The amide I envelope was curve-fitted as a sum of Voigt peaks in Fityk [40], where the initial guess of the positions of these peaks were determined using the minima (in the second-derivative spectra) and the maxima (in the fourth-derivative spectra). The goodness of the fit was further determined by comparing the second derivative of the fitted spectra with that of the original spectra.

**Analytical ultracentrifugation (AUC).** AUC sedimentation velocity (AUC-SV) experiments were performed using a Beckman ProteomeLab XL-I analytical ultracentrifuge (Beckman, Indianapolis, IN, US) with a rotor An-50Ti. E protein samples were reconstituted in 5 mM myristyl sulfobetaine (C14SB, Sigma), 50 mM Tris pH 7.3, and 100 mM NaCl, in presence of 29.4% (*v*/*v*) D_2_O to eliminate detergent buoyancy. The samples were centrifuged at 50,000 rpm in epon 2-sector centerpiece AUC cells with quartz windows. Absorbance profiles at 230 and 280 nm were collected every 10 min for 15 h. Sedimentation profiles were analyzed in SEDFIT using the c (s) model [41] and plotted with GUSSI [42].

S-values corresponding to monomer and pentamer in C14SB micelles were predicted considering the properties of detergent, protein, and buffer composition. The values for molecular weight (MW) and partial specific volume of C14SB was 363.6 Da and 0.965–0.978 mL/g (based on our density-matching data), respectively. A value of 0–10 was used for the aggregation number of C14SB, since the buffer was density-matched using 29.4% D_2_O. The MW of the protein calculated from the amino acid sequence is 8542 Da and 9081 Da for E-FL and His-E-TR, respectively, and the partial specific volume is 0.765 mL/g and 0.7543 mL/g for E-FL and His-E-TR, respectively (calculated using Sednterp software [43]). The density and viscosity of the buffer (50 mM Tris, 100 mM NaCl and 29.4% D_2_O) was ρ = 1.0353 g/mL and η = 1.0997 cP (calculated using Sednterp software), respectively. When the detergent density was balanced using D_2_O, it did not contribute to the protein–detergent complex’s buoyant MW, and, thus, we can consider the detergent bound to be effectively zero. Using this value (detergent bound = 0) and the lowest estimate of C14SB specific volume (ν_D_), weight of the protein–detergent micellar complex (M_C_), mass fraction of the detergent (δ_D_), lowest specific volume of the complex (ν_C_), buoyant mass (Mb), and higher boundary for S value can be calculated (Table 1). A lower boundary for S value for the monomer and pentamer species of E protein can be calculated by including some bound detergent (e.g., 10 per monomer) and using the highest estimate for detergent specific volume: 0.44 S (monomer) and 1.89 S (pentamer) for His-E-TR and 0.40 S (monomer) and 1.71 S (pentamer) for E-FL (last column in Table 1).

AUC sedimentation equilibrium (AUC-SE) experiments were performed for His-E-TR and E-FL samples using the same instrument, rotor and buffer conditions as the AUC-SV samples. For each sample, three concentrations were prepared (30, 50, and 80 µM) and centrifuged at four speeds (23,000, 28,000, 34,500 and 42,000 rpm) in 6-sector Epon centerpiece AUC cells with quartz windows. Absorbance profile at 280 nm was collected after 24 h equilibration at each speed (equilibrium was confirmed by comparing scans obtained at 30 min interval). Multi-speed sedimentation profiles collected at equilibrium was fitted to various self-association models in SEDPHAT and plotted in GUSSI [42,44]. The species population plot was drawn in concentration scale.

## 5. Conclusions

Herein, we provide evidence that the SARS-CoV-2 E protein changes its conformation (secondary structure) in lipid membranes triggered by oligomer formation. The formation of the β-structure occurs in ~15 amino acids located at the C-terminal extramembrane domain, likely where the β-strands are predicted to form. We hypothesize that this β-structure formation is an integral characteristic of the channel structure in the full-length E protein and may have a role in membrane scission during virion formation.

## Figures and Tables

**Table 1:** Prediction of range of S values for E protein monomers and pentamers.

Sample	f/f_0_^1^	M_C_ (Da) ^2^	δ_D_^3^	ν_C_ (mL/g) ^4^	Mb (Da) ^5^	Diam. (nm) ^6^	S ^7^	S-Range ^8^
His-E-TR monomer	2.2	9081 + 30,179 = 39,260	0.000	0.754	1989	2.8	0.52	0.44–0.52
His-E-TR pentamer	1.7	45,405 + 30,179 = 75,584	0.000	0.754	9947	4.8	1.97	1.89–1.97
E-FL monomer	2.2	8542 + 30,179 = 38,721	0.000	0.765	1777	2.8	0.47	0.40–0.47
E-FL pentamer	1.7	42,710 + 30,179 = 72,889	0.000	0.765	8883	4.7	1.78	1.71–1.78

^1^ The frictional ratio (f/f_0_) was taken from SEDFIT c (s) analysis results. ^2^ Molecular weight of the protein–detergent complex (M_C_). ^3^ The density of the buffer is matched with that of the detergent using D_2_O, so the mass fraction of the detergent (δ_D_) now reflects any density mismatch instead of amount of detergent physically bound. A value of zero was used for calculating the higher bound of S value. ^4^ The specific volume (1/density) of the protein–detergent complex. ^5^ The buoyant molecular weight of the protein–detergent complex. ^6^ The diameter of the protein–detergent complex is calculated assuming a spherical shape. ^7^ The lower estimate of S value. ^8^ The range of S value assuming density mismatch equivalent to 0–10 detergent molecules bound.

## Data Availability

Not applicable.
